# Remineralization potential of phosphorylated chitosan and silver diamine fluoride in comparison to sodium fluoride varnish: invitro study

**DOI:** 10.1007/s40368-023-00794-2

**Published:** 2023-04-04

**Authors:** Y. Mohamed, R. Ashraf

**Affiliations:** 1 Pediatric Dentistry Department, Faculty of Dentistry, Ahram Canadian University, Giza, Egypt; 2 Prosthetic Dentistry Department, Faculty of Dentistry, King Salman International University, El Tur, South Sinai Egypt

**Keywords:** Silver diamine fluoride, Phosphorylated chitosan, Biomimetic enamel remineralization, Enamel microhardness, Scanning electron microscopy

## Abstract

**Purpose:**

The purpose of this study was to evaluate and compare the remineralization potential of phosphorylated chitosan nanoparticles (Pchi) and silver diamine fluoride (SDF) compared to sodium fluoride varnish (NaF) on microhardness of artificial carious lesions in a biomimetic minimally invasive approach that is being regarded as the future of preventive dentistry.

**Methods:**

The sample size included 40 intact extracted maxillary anterior human teeth. Baseline microhardness was recorded using Vickers hardness test and energy-dispersive X-ray spectroscopy (EDX). Artificial caries-like lesions were created on the exposed enamel by suspending all teeth in demineralizing solution for 10 days in a temperature of 37 °C and then the hardness and EDX were remeasured. Samples were then divided into four main groups: Group A (positive control group) *n* = 10, treated with NaF, Group B *n* = 10, treated with SDF, Group C *n* = 10, treated with Pchi and Group D (negative control group) *n* = 10 that received no treatment. After treatment, samples were incubated in artificial saliva solution at 37 °C in for 10 days and then reassessed. Data were then recorded, tabulated, and statistically analyzed using Kruskal–Wallis test and Wilcoxon signed test. Scanning electron microscope (SEM) was used to analyze the morphological changes of enamel surface after treatment.

**Results:**

Groups B and C showed the highest calcium (Ca) and phosphate (P) content as well as hardness values, while group B had the highest percentage of fluoride. SEM revealed a smooth layer of mineral formed on the surface of enamel for both groups.

**Conclusion:**

Pchi and SDF showed the highest increase in enamel microhardness and remineralization potential.

**Clinical relevance:**

The minimally invasive approach for remineralization could be enhanced using SDF and Pchi.

## Background

Dental caries is still a global health problem affecting many children. It is considered as the most common chronic disease that affects children. This research in dentistry was directed toward minimally invasive approaches for treatment, especially that dental procedures are unfavorable for children in addition to their high cost (Bagramian et al. 2009).

The early stage of dental caries is the mineral loss from the enamel surface subjected to caries. In the old days, the drill and fill technique was used for treatment; nowadays, especially with the evolution of remineralizing agents that can recover the mineral loss, it is being regarded as a very convenient solution and a preventive measure to control the demineralization process instead of invasive techniques that were used before (Arifa et al. 2019).

The most prevalent therapy for many years was the use of fluoride, which can rebuild partially dissolved crystals, supplied in the form of topical gel or varnish that was applied to arrest active dental caries by depositing calcium fluoride that is not readily soluble and can act as a fluoride reservoir to enhance enamel remineralization, increasing the speed of the remineralization process and the mineral content of early carious lesions. This therapy is a relatively low-cost and easily performed treatment. Fluoride was also incorporated in the water and dietary supplements for the same purpose (Gao et al. 2016; Ten Cate and Buzalaf 2019).

However, despite the remarkable beneficial effect of fluoride on enamel remineralization, dental fluorosis induced by excessive intake of fluoride could not be ignored. Fluoride adversely affects tooth development either through interactions with the developing ameloblasts or the intracellular matrix, if the amount of fluoride is not adjusted. Therefore, the administration of fluoride-containing dental products in children should be reviewed carefully (Rubio et al. 2017).

One of the derivatives of fluoride is sodium fluoride (NaF) varnish that is applied topically in the concentration of 5%. The fluoride intake by the tooth surface is dependent on its concentration, and the increase in the contact time between fluoride and tooth structure; thus, it is necessary for the patient to cooperate due to the need for multiple application sessions annually.

Several alternatives to fluoride emerged in the dental market. together with the further understanding of biomineralization process of dental hard tissue that made science eager to develop biomimetic remineralization strategies by mimicking the crystallization pathway with amorphous precursors of hydroxyapatite (HAP) (Cao et al. 2015).

The biomimetic approach relies on the production of enamel-mirroring material and is regarded as the future of preventive dentistry as correction of enamel defects, with the difficulties faced upon using restorative materials due to weak mechanical strength and poor retention (Zhang et al. 2014).

Moreover, chitosan, which is a linear co-polymer of glucosamine and *N*-acetyl glucosamine obtained by *N*-deacetylation of chitin, is one of the widely used materials in tissue engineering. Chitosan and its derivatives have emerged as a new class of novel biomaterials, due to their versatile biological activity, excellent biocompatibility, antibacterial and remineralizing effects on tooth structure. These have positive charges, which accumulate on the cell walls of the bacteria, providing bactericidal and bacteriostatic property that would be of great importance in combating the effect of *Streptococcus mutans*, which is most common bacteria associated with dental caries (Satitsri and Muanprasat 2020).

Among these derivatives is phosphorylated chitosan (Pchi), which exhibits metal chelating properties. The chelating ability of the phosphate groups of Pchi for calcium ions immobilizes Pchi molecules on the tooth surface and helps in binding calcium ions to form nucleating sites for remineralizing enamel subsurface lesion based on the biomimetic strategy (Chellapandian et al. 2020).

Silver diamine fluoride (SDF) (38%) is another form of professionally applied fluoride. The first SDF product was approved in the USA by the Food and Drug Administration (FDA) in 2014, and now SDF is of high popularity for its safety and effective control and arrest of caries (Horst 2018).

It is a colorless liquid containing silver particles and 38% (44,800 ppm) fluoride ions (which is the highest fluoride concentration available for dental use) that at pH 10 is 25% silver, 8% ammonia, 5% fluoride and 62% water. It is the first material to combine the remineralizing capacity of sodium fluoride with the antibacterial effect of silver nitrate (Zhao et al. 2018; Chibinski et al. 2017).

Nevertheless, silver component in SDF may cause black staining of the carious lesion, which may not be accepted by the patients or their parents. Therefore, the choice of effective no-fluoride anti-caries agents are being explored. Also, claimed limitations of those therapies is that remineralization takes place predominantly on the lesion surface. This surface precipitation will probably fill the superficial pores and block pathways to the lesion body, giving rise to a restriction in complete lesion consolidation and decreased hardness of the tooth surface (Magno et al. 2019).

Although many research works have discussed the remineralizing modalities, to our knowledge, studies comparing the remineralizing effect of phosphorylated chitosan to silver diamine fluoride are very limited.

Thus, use of NaF, Pchi and SDF , in the form of varnish that is directly applied on the tooth surface without the need of drilling into the tooth structure, facilitating the dental procedure and aiming to remineralize the tooth surface, is being investigated in this study. By evaluation of enamel microhardness using Vickers microhardness test, the surface morphology of the samples will be further investigated and observed via scanning electron microscopy (SEM) with EDEX to quantify the amount of calcium deposited on the surface.

Null hypothesis is that Pchi and SDF would enhance the remineralization of enamel and thus enamel microhardness.

## Materials and methods

### Remineralizing agents

#### a. Phosphorylated chitosan hydrogel

One gram (g) of chitosan powder, 5 g of urea, and 10 mL of phosphoric acid were added to 40 mL of dimethylformamide. The mixture was stirred continuously at 150 °C for 1 hour (h) by heating in an oil bath. Upon cooling to room temperature, the solution was filtered, the precipitate thoroughly washed with distilled water and anhydrous ethanol and then dried. 0.5 % phosphorylated chitosan gel was prepared. Carboxymethyl cellulose (CMC) was used as a gelling agent. First, a portion of CMC was dissolved in15 ml distilled water. Pchi was then added and dissolved well to form a 0.5% concentration. The gel was stored at 2–8 °C for further application (Mikušová and Mikuš 2021).

#### b. Silver diamine fluoride (SDF)

It was purchased from Advantage Arrest, USA, with 38% (44,800 ppm) fluoride ion that at pH 10 is 25% silver, 8% ammonia, 5% fluoride, and 62% water.

#### c. Sodium flouride varnish

It was purchased from 3M ESPE, Seefeld, Germany with 5% sodium fluoride (22,600 ppm fluoride), sweetened with xylitol and containing a tricalcium phosphate ingredient (TCP).

#### d. Artificial saliva

It contains distilled water (700 ml), CaOH_2_ (1.56 mM), KCl (150.00 mM), HCl (36.00 mM), H3PO4 (0.88 mM), buffer (99.7 mM), pH 7.2).

### Preparation of samples

The study protocol was approved by the ethical committee of Cairo University with approval number 1522. A total number of 40 intact extracted maxillary anterior human teeth were collected from the institute of diabetes for the study. Teeth were cleaned using ultrasonic scaler and stored in artificial saliva. Enamel slabs were cut from the buccal surfaces of each tooth under sufficient water flow and then mounted in acrylic resin. They were stored in artificial saliva to simulate the environment of the oral cavity. The storage media was changed weekly to prevent any bacterial growth (Hegde and Moany 2012; El Hagry et al. 2021).

### Demineralization of samples

Artificial caries-like lesions were created on the exposed enamel by suspending all teeth in demineralizing solution for 10 days at a temperature of 37 °C. The solution was prepared by mixing 5 l of distilled water and 2.205 g of calcium chloride (CaCl_2_) with 2.041 g potassium dihydrogen phosphate (KH_2_ PO_4_). The solution was renewed daily (Bajaj et al. 2016).

After demineralization, they were stored in artificial saliva till testing (Ashraf and Aidaros 2021).

### Sample grouping

- Group A: Positive control group, *n* = 10. Samples were coated with sodium fluoride, followed by incubation at 37 °C in artificial saliva solution for 10 days.
- Group B: *n* = 10. Samples were coated with silver diamine fluoride, followed by incubation at 37 °C in artificial saliva solution for 10 days.
- Group C: *n* = 10. Samples were coated with phosphorylated chitosan, followed by incubation at 37 °C in artificial saliva solution for 10 days.
- Group D: Negative control group, *n* = 10. Samples were coated and incubated at 37 °C in artificial saliva solution for 10 days.

### Testing of samples

#### a. Enamel microhardness test

Samples were assessed for microhardness values of enamel surface using micro-Vicker’s hardness machine (Prisma, Germany) at 100 g for 5 s. The hardness of the samples was measured before demineralization (T1), after demineralization (T2) and after remineralization (T3).

T1: first time before demineralization.

T2: second time after demineralization.

T3: third time after remineralization.

#### b. Assessment of morphology

i. Scanning electron microscopy (SEM)
  SEM was used for assessment of morphological changes at the enamel surfaces after the remineralization procedures at 1000×, 2000× and 5000× after metallic coating of the samples at 20,000 kV.
ii. Energy-dispersive X-ray analysis (EDX assessment)
  Surface analysis (for presence or absence of minerals and their percentage, especially calcium and phosphorus) by energy-dispersive analysis X-ray and EDX spectrum images. Data were recorded within an average of 6 min for each specimen.

## Results

*Statistical analysis* Data were explored for normality using Kolmogorov–Smirnov and Shapiro–Wilk tests. Elemental analysis weight % data showed normal distribution, so one-way ANOVA was used to compare between tested groups. Multiple comparisons with Tukey’s HSD were performed for pairwise comparisons. Ca/P ratio showed non-parametric distribution, so Kruskal–Wallis test was used to compare between different tested groups.

The significance level was set at *P* ≤ 0.05. Statistical analysis was performed with IBM SPSS Statistics for Windows, version 26.0. Armonk, NY, IBM Corp.

### Energy-dispersive X-ray element analysis (EDX)

**Fig. 1:**
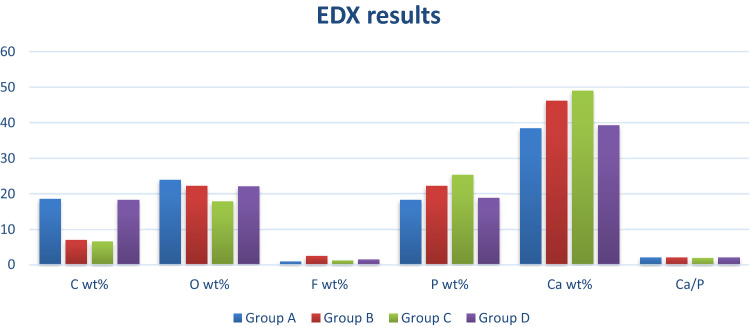
Bar chart showing the mean elemental weight % for different tested groups

Within group comparison results of the present study show that after remineralization, the carbon (C) content was significantly higher for groups A and D than groups B and C, while the oxygen (O) content for group C was significantly lower than that of other groups. Fluoride (F) content was significantly higher for group B than groups C and D. Group A showed the lowest F percentages in all groups. Calcium (Ca)/phosphate (P) ratio increased significantly in all groups with no statistically significant difference among them as represented in Table 1 and Fig 1.

**Table 1:** Mean, standard deviation (SD) of elemental weight % of carbon (C), oxygen (O), fluoride (F), phosphate (P), calcium (Ca) and calcium to phosphate ratio (Ca/P) for different tested groups

	C wt%		O wt%		F wt%		Ph wt%		Ca wt%		Ca/P	
	Mean	SD	Mean	SD	Mean	SD	Mean	SD	Mean	SD	Mean	SD
Group A	18.5a	4	23.9a	1.2	0.9c	0.3	18.3c	2.1	38.4c	1.8	2.1a	0.2
Group B	7b	1.6	22.2a	1.3	2.5a	0.1	22.2b	1.4	46.2b	1.3	2.1a	0.1
Group C	6.6b	3	17.8b	1.1	1.2b	0.1	25.3a	0.8	49.0a	2	1.9a	0.1
Group D	18.3a	1.8	22.1a	1.5	1.4b	0.1	18.8c	0.8	39.2c	1	2.1a	0.1
*p* value	< 0.001*		< 0.001*		< 0.001*		< 0.001*		< 0.001*		0.078 NS	

Different letters within a column indicates significant difference between groups (*p *< 0.05, adjusted with Bonferroni correction for multiple comparisons)

*significant, NS=non significance

### Microhardness of enamel surface (VH)

**Fig. 2:**
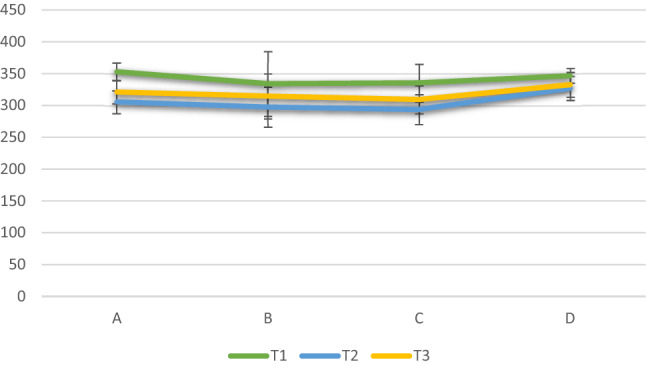
Bar chart showing the mean VH for different tested groups

For group A, significant decrease in the microhardness resulted at T2, followed by a significant increase at T3. However, the VH values at T3 were still significantly lower than T1. For group B, C and D, significant decrease in the microhardness resulted at T2, followed by an insignificant increase at T3. Thus, group A and B were the only ones that showed insignificant values between T1 and T3, as represented in Table 2 and Fig. 2.

**Table 2:** Mean and standard deviation (SD) of VH for different tested groups before demineralization (T1), after demineralization (T2), and after remineralization (T3)

Time	T1 (before demineralization)		T2 (after demineralization)		T3 (after remineralization)		*p*-value
Group	Mean	SD	Mean	SD	Mean	SD	
A	352.9^a^	13.5	305.6^c^	17.7	321.1^b^	18	< 0.001*
B	339.5^a^	44.9	297.5^b^	31.7	317.5^ab^	31.2	0.011*
C	335.5^a^	29.1	293.7^b^	23.8	315.4^ab^	20.2	< 0.001*
D	346.8^a^	11.2	327.1^b^	18.6	332.6^b^	19.3	0.044 NS
*p*-value	0.354 NS		0.062 NS		0.105 NS		

Different letters within a column indicate significant difference between groups (*p *< 0.05, adjusted with Bonferroni correction for multiple comparisons)

*significant, NS=non significance

### Scanning electron microscope (SEM)

**Fig. 3:**
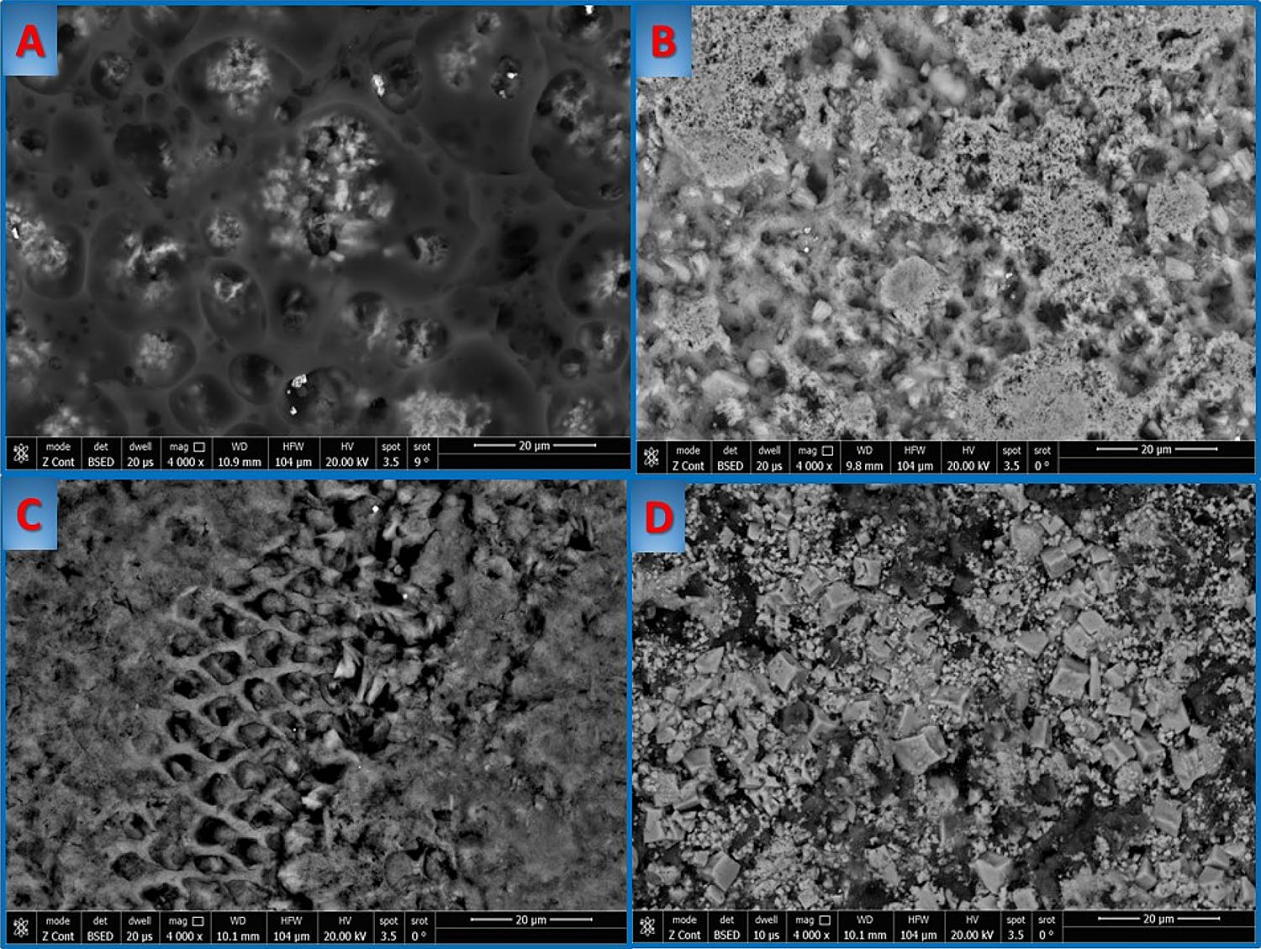
SEM image of groups. **A** Enamel surface treated with sodium flouride varnish, **B** enamel surface treated with silver diamine flouride, **C** enamel surface treated with phosphorylated chitosan, **D** untreated enamel surface

The surface morphology of the demineralized enamel sections after remineralization is shown in Fig. 3. Scanning electron microscopy (SEM) at 1000×, 2000× and 4000× was used for assessment of morphological changes at the enamel surfaces after the remineralization procedures. Figure 3A represents the surface of enamel treated with NaF varnish, where a layer of mineral is formed on the surface covered by a layer of varnish that gives two contrasting colors. In Fig. 3B, where the surface was treated with SDF, the surface was relatively dense and intact compared with other groups. A particular feature was observed on the homogenous enamel surface, where reduction in porosities, a dense layer of remineralization surface, and white spots representing silver were spotted in the sample. Regarding Fig. 3C, the surface showed that remineralization with Pchi resulted in deposition of mineral particles on the enamel, showing a comparatively smooth surface that unfortunately was surrounded by a layer of less remineralized crystals. Figure 3D shows a high pattern of corrosion, represented by a loss of enamel rod peripheries with intact rod cores, leading to widening in interprismatic distance, while some areas showed microporosities due to loss of rod cores and relatively intact peripheries.

## Discussion

Different methods to determine the effectiveness of remineralizing agents on artificial caries lesions were used. Mineral loss and uptake were measured by determining the mineral content on enamel surfaces using EDX. Vickers hardness test was used to assess the hardness of remineralized surfaces. Morphological changes on the surface of the remineralized tooth can also be detected using the SEM, having the advantage to characterize the morphological features of remineralization effects in initial artificial caries lesions (Arnold et al. 2007).

In this present study, assessment of enamel remineralization was conducted using conventional application NaF compared to SDF and Pchi. EDX assessed mineral weight percentage in different groups, which was used to clarify the images of SEM, since the main components of hydroxyapatite are calcium (Ca) and phosphate (Po4). Therefore, these elements were the main objects of this study. Moreover, monitoring Ca/Po4 ratio changes was done for all samples in the four groups (Vicente et al. 2017).

The results of this study agreed with the null hypothesis in which SDF and phosphorylated chitosan were found to enhance enamel remineralization.

During remineralization, ion substitution in forming apatite takes place, which includes substitution of calcium ions with magnesium and sodium, substitution of hydroxyl sites with fluoride and chloride, and substitution of both phosphate and hydroxyl sites with carbonate. Thus, minerals other than Ca and P could have a great influence on the remineralization process, while considerable variation in apatite properties could occur, for example, carbonate substitution increases solubility of apatite and fluoride substitution decreases its solubility (Nanci 2017).

The evaluation of the remineralization potential in terms of mineral content fills the gap in knowledge about the type of action of both SDF and Pchi on enamel by comparing their remineralizing capacity to the regularly used NaF gel. Mineralization in general begins with the formation of hydroxy apatite (HA) crystals within matrix vesicles, followed by propagation of HA through the membrane into the ECM. When the accumulation of calcium and phosphate exceeds the solubility point for calcium phosphate, deposition of HA occurs within the matrix vesicles (Orimo 2010).

Upon monitoring the weight percentage of minerals of the groups A, B, C and D, there was a significant decrease in the carbon content (C) with SDF and Pchi groups in comparison to NaF and the negative control group. The oxygen (O) content was higher in the SDF group in comparison to the Pchi groups that has shown significant decrease in the (O) content. This would favor the mechanism of action of SDF that relies on silver reaction with oxygen, phosphorus, and sulfur present in the demineralized lesion, leading to the formation of silver phosphate, silver oxide, and silver sulfide (Mei et al. 2018; Li et al. 2019).

The highest F content was found with SDF, followed by Pchi groups; they were significantly higher than NaF gel that would rather explain the high hardness number achieved by these two groups in comparison to groups A and C. Regarding the Ca, P and Ca/P ratio in all groups after remineralization, the percentage increase of Ca and p in Pchi group was higher than that of the SDF group, which was also higher than that of the NaF group with significant differences between them.

This could because SDF promotes absorption of calcium, leading to inhibition of calcium dissolution from enamel. Silver ions in the SDF, responsible for the antimicrobial action, react with hydroxyapatite crystals, resulting in the precipitation of silver phosphate that forms an insoluble hard layer on the enamel surface. The remineralizing capacity of SDF is also promoted by the presence of fluoride ions found in the solution. In the presence of calcium and phosphate, fluoride forms fluorapatite crystals and calcium fluoride; this increases the mineral density and hardness (Ten Cate and Buzalaf 2019; Milgrom et al. 2018).

Additionally, chitosan attracts phosphate ions from saliva through the electrostatic forces of the NH_3_ groups. Also, the functional carboxyl that is present on the surfaces of chitosan is responsible for effective spontaneous apatite nucleation, in an ordered orientation over the surface (De Carvalho et al. 2011).

This might explain the hardness test results that indicated that groups B and C experienced increase in hardness values after remineralization (T3) to the extent of reaching the same value of hardness before remineralization(T1), and both were significantly higher than the hardness after demineralization (T2). Unlikely in group A, although the remineralization with NaF varnish increased the surface hardness of the specimen, it did not actually return to the value before demineralization (T1).

The results are in accordance with Yihong et al. (2019) and Noaman et al. (2020), as they agreed that chitosan and SDF are more effective in remineralization than NaF varnish. On the other hand, our research is in disagreement with Zhang et al. (2014) that found the remineralizing effect of Pchi–ACP was similar to that of fluoride.

Environmental scanning electron microscope (SEM) was further used to evaluate and record the ultrastructural changes of the enamel surface. As it is a non-destructive characterization technique, which requires little or no sample preparation, the samples treated with SDF showed that the prismatic pattern displayed thick lines of remineralization along the prismatic borders. Samples treated with Pchi revealed improvements of the enamel ultrastructure, although certain areas of demineralization were evident along with the porosities but specimens manifested a smoother enamel surface with decreased number and size of previously formed pores compared to demineralized enamel, indicating an increase in mineral density. Accordingly, SDF and Pchi helped the most in the biomineralization process of enamel tissue and controlled the mineral crystallites better than NaF.

The limitations of the study were mainly related to time constraints where the follow-up of remineralization ability after a long time would have given more clear results regarding the remineralization potential of each protocol. Also, the application of remineralizing agents for more than three times would lead to more pronounced effect.

Limitations could be attributed to the insufficient sample size for statistical measurements. Lack of enough research on Pchi also limited the ability to compare it to other remineralizing agents. The study was carried on extracted teeth and further research in vivo would enrich the results.

## Conclusions

Considering the limitations of the present study, the following conclusions can be made:

- Pchi and SDF would beneficially aid in enamel remineralization.
- Phosphorylated chitosan aids in the formation of enamel patterns that mimics the prismatic orientation of enamel rods, which would help in enamel tissue regeneration.
- NaF varnish is still a valid approach for remineralization.
- Further studies should be carried out to substantiate the findings of this study.

## Data Availability

The datasets used and/or analyzed during the current study available from the corresponding author on reasonable request.
